# The Outcome and Economic Viability of Embryo Production Using IVF and SOV Techniques in the Wagyu Breed of Cattle

**DOI:** 10.3390/vetsci7020058

**Published:** 2020-05-01

**Authors:** Fernanda L. Facioli, Flávia De Marchi, Mariana G. Marques, Paulo R. P. Michelon, Eraldo L. Zanella, Kyle C. Caires, Jerry J. Reeves, Ricardo Zanella

**Affiliations:** 1 Faculdade de Agronomia e Medicina Veterinária, Curso de Medicina Veterinária, Universidade de Passo Fundo, Passo Fundo, RS 99052-900, Brazil; 147652@upf.br (F.L.F.); 144320@upf.br (F.D.M.); ezanella@upf.br (E.L.Z.); 2 Programa de Mestrado em BioExperimentação, Universidade de Passo Fundo, Passo Fundo, RS 99052-900, Brazil; 3 Programa de Pós-Graduação em Produção e Sanidade Animal, Instituto Federal Catarinense, Concórdia, SC 89703-720, Brazil; marigroke@gmail.com; 4 Laboratório de Sanidade Animal, Embrapa Suínos e Aves, Concórdia, SC 89715-899, Brazil; 5 Médico Veterinário Autônomo, Passo Fundo, RS 99052-321, Brazil; paulomichelon14@yahoo.com.br; 6 Department of Human Nutrition, Food and Animal Sciences, University of Hawaii, Manoa, HI 96822, USA; kccaires@hawaii.edu; 7 Bar-R Cattle Company, Pullman, WA 99163, USA; jreeves@colfax.com

**Keywords:** superovulation, in vitro fertilization, Wagyu

## Abstract

The Japanese black cattle breed (Wagyu) has an improved metabolism, which allows them to have a higher marbling score when compared with other cattle breeds. However, this may affect other aspects of the animal’s physiology, including hormone secretion and their reproductive success, such as their response to synchronization protocols and embryo production. Therefore, the objectives of this study were to test a superovulation protocol (SOV) developed with low doses of FSH and to evaluate the outcome and economic viability of embryo production using the SOV and in vitro fertilization (IVF) approaches in the Wagyu cattle breed. For that, ten Wagyu cows were submitted to five SOVs over a period of 15 months using a standard protocol: CIDR + 3 mg estradiol benzoate (D0), 35 mg FSH (Folltropin^®^) a.m. and p.m. (D4), 35 mg Folltropin^®^ a.m. and 20 mg p.m. (D5), 20 mg Folltropin^®^ a.m. and 10 mg p.m. (D6), 10 mg Folltropin^®^ and 0.5 mg cloprostenol, both a.m. and p.m., + CIDR removal (D7), 0.05 mg GnRH + insemination 12 and 24 h after (D8) and embryo collection + 0.5 mg of cloprostenol (D16). Thirty days after each SOV, a follicular aspiration was conducted to produce IVF embryos without any pre-synchronization using standard semen in the same group of animals. The average number of embryos produced was 7.63 ± 5.61 (SOV) and 4.52 ± 2.44 (IVF) (*p* = 0.303). There was no significant correlation between the number of embryos produced by the different techniques (SOV and IVF), indicating that cows that respond well to SOV did not respond well to IVF and vice versa (r = 0.379, *p* = 0.529). The total cost of each embryo produced by SOV was R$215.00 and R$410.00 for IVF. Therefore, cows that produce less than five embryos by SOV are not economically viable due their lack of response to FSH, and the use of IVF in those animals may be more effective.

## 1. Introduction

The Japanese black cattle breed, also known as Wagyu, was developed from different crosses among Asian cattle breeds. There are three major black strains of Wagyu (Tajima, Shimane and Kedaka), which were developed due to regional geographic isolation in Japan, comprising 90% black cattle with the remainder being red [1,2].

The Tajima line originated from the Hyogo prefecture where these animals were traditionally used to pull carts and ploughs; accordingly, these cattle developed larger forequarters and lighter hindquarters. Generally, the Tajima-line cattle are small-framed, with slower growth rates, but they produce excellent meat quality, being prized for their superior marbling and for having a large eye muscle relative to their carcass weight. The Shimane line originated in the Okayama prefecture and are known for being animals with a medium frame size, average growth rates and good meat quality. Finally, the Kedaka line, stemming from the Tottori prefecture, were mainly used as pack animals to serve the grain industry and, as a result, were selectively bred to have straight, strong back lines, with good growth rates and a large size. While the Kedaka strain is the best for milking ability, their meat quality and carcass characteristics remain highly variable [1]. 

Initially, Wagyu cattle were selected as draft animals based on their physical endurance for their use in agriculture [3,4,5]. This type of selection indirectly selected animals with higher intramuscular fat content, also known as marbling, which provided a readily available energy source [1,4,5,6,7,8]. The higher concentration of marbling is associated with the presence of a higher percentage of monounsaturated fatty acids (MUFA) in Wagyu beef, when compared with other cattle breeds; one example is oleic acid, a MUFA that is distributed inside of the muscle fibers [4,5,6]. The higher percentage of MUFA is also correlated with a lower melting point for the fat, which contributes to improvements in tenderness, beef flavor and overall eating quality. The emphasis on producing beef containing fat with a lower melting point has become an important consideration in the beef industry, especially in Japanese black cattle [8]. The demand for well-marbled beef with higher concentrations of beneficial fatty acids (such as omega-3 and omega-6) is great, and therefore, Wagyu cattle offer tremendous value in various segments of the meat-producing chain [3,9,10,11,12].

The last export of Wagyu sperm and embryos from Japan occurred in 1999, and to that end, Japanese cattle producers have made efforts to keep Wagyu genetics inside Japan to protect their beef brand. As a result, very little genetic diversity exists in the Wagyu breed of cattle outside of Japan, which creates challenges from an animal breeding standpoint [2]. The reduced numbers in the effective population size, resulted from mating between relatives, often leads to the production of highly inbred animals with reduced reproductive success [6,13]. 

There is genetic evidence in the Wagyu cattle breed associated with variable levels of responses to superovulation protocols. Sugimoto et al. [14] have identified an association of a single nucleotide polymorphism (SNP) in the glutamate ionotropic receptor AMPA type subunit 1 (*GRIA1*) with ovulation rates in Wagyu cattle. Hirayama et al. [15] have further characterized variations in the allele frequency of *GRIA1* among different lines of Wagyu, indicating an effect of the genetic background on the responses to superovulation. Therefore, the selection of animals with the allele favorable to a superovulation protocol (SOV) response might improve reproductive success in Wagyu cattle.

For genetic improvement in cattle, artificial insemination (AI) is the preferred method used by breeders, due to the possibility to use semen from genetically superior animals [16]. The most advanced reproductive biotechnologies, such as superovulation, embryo transfer and in vitro fertilization techniques [17], together with the use of sexed semen [18], are being extensively used to increase the Wagyu population while limiting the amount of inbreeding [13,19]. 

Limited information is available regarding the response of the Wagyu cattle to SOV protocols. In China, the superovulation protocols with the use of 400 mg of Folltropin have produced an acceptable number of transferable embryos per collection per cow [20]. However, not much is known about the success of the reproductive characteristics of the Wagyu breed in Brazil, South America—a country with tropical and subtropical climates. Additionally, since Wagyu cattle are well known to have a higher metabolism related to the increased intramuscular fat accumulation, it is speculated that their endocrine system was also altered with the potential to negatively affect embryo quality and production following different advanced reproductive technologies. 

Those challenges, coupled with high inbreeding rates in the cattle outside of Japan, are likely responsible for their inherently lower reproductive success; therefore, we strongly feel that embryo transfer offers a potential means to rapidly increase the propagation of Wagyu cattle, such that greater rates of genetic improvement can occur. Hormone costs and the efficacy of the SOV technique are key components involved with successful use of a SOV in Brazil. The objectives of this study were to test a superovulation protocol (SOV) using a reduced dose of FSH to evaluate the outcome of embryo production in Wagyu cows and to determine the economic viability using this new SOV protocol compared to an in vitro fertilization (IVF) approach.

## 2. Materials and Methods

### 2.1. Animals

Ten cyclic, nonlactating Wagyu cows (average age of 26 months; min = 18 months, max = 48 months) of mainly the Tajima line (high marbling line) were used in the present study. The cows were maintained in a semi-extensive breeding system in the Northwest Region of the Rio Grande do Sul State, Brazil, at 631 m above sea level, (Latitude: 27°41′20″ south, Longitude: 51°46′6″ west). Animals were grazed on native grasslands and provided mineral supplementations with MUB Beef Perform [21]. This experiment was conducted following the principles set down by the animal care procedures of the Ethics Committee on Animal Utilization of the University of Passo Fundo CEUA-013/2019.

### 2.2. Superovulation Protocol and Follicular Aspiration

**Figure 1:**
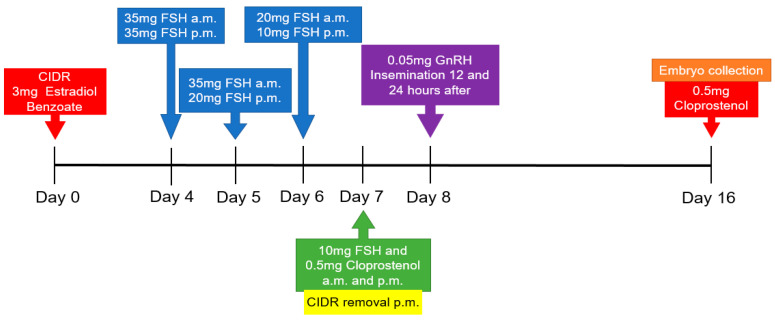
Superovulation (SOV) protocol used in Wagyu cows.

During a period of 15 months, the animals were submitted to five superovulations (SOVs) using a standard protocol developed and tested for the Wagyu cattle breed by our group (Figure 1). The protocol consisted of the use of CIDR + 3 mg of estradiol benzoate on day 0; on day 4 to day 7, animals received decreasing doses of FSH (Folltropin^®^-V, Vetoquinol, Fort Worth, TX, USA). Day 4, 35 mg of FSH in the morning and 35 mg in the afternoon; on day 5, animals received 35 mg of Folltropin^®^ in the morning and 20 mg in the afternoon; on day 6, animals received 20 mg of Folltropin^®^ in the morning and 10mg in the afternoon; on day 7, animals received 10mg of Folltropin^®^ and 0.5 mg of cloprostenol, both in the morning and afternoon and, also in the afternoon, the CIDR was removed. On day 8, 0.05 mg of gonadorelin acetate (GnRH) was administered, and insemination was conducted 12 and 24 h after its injection. On day 16 of the protocol, the embryo collection was conducted, followed by administration of 0.5 mg of cloprostenol. Thirty days after each SOV, a follicular aspiration from each cow (without use of pre-synchronization) was conducted by two different commercial laboratories. Oocytes collected by aspiration were used to produce in vitro fertilization (IVF) embryos using standard semen. A highly skilled technician specific to each laboratory produced the IVF embryos, which were classified according to the criteria set by the International Embryo Technology Society [22]. Numbers of IVF embryos produced per cow per laboratory were not significantly different, indicating the consistency of results between the two laboratories.

### 2.3. Statistical Analysis

Categorical variables were summarized using absolute frequency and percentage values. Continuous variables were summarized using the mean and standard error of the mean (SEM) values. The data normalities were verified using the Shapiro-Wilk Test; further, the Student *t*-test was used to compare the means of embryo produced between SOV and IVF procedures. The Pearson correlation coefficient was estimated between the number of embryos produced per cow, treatment and time. Finally, the total cost for each procedure were analyzed. The results were considered significantly different at *p* < 0.05.

## 3. Results

**Figure 2:**
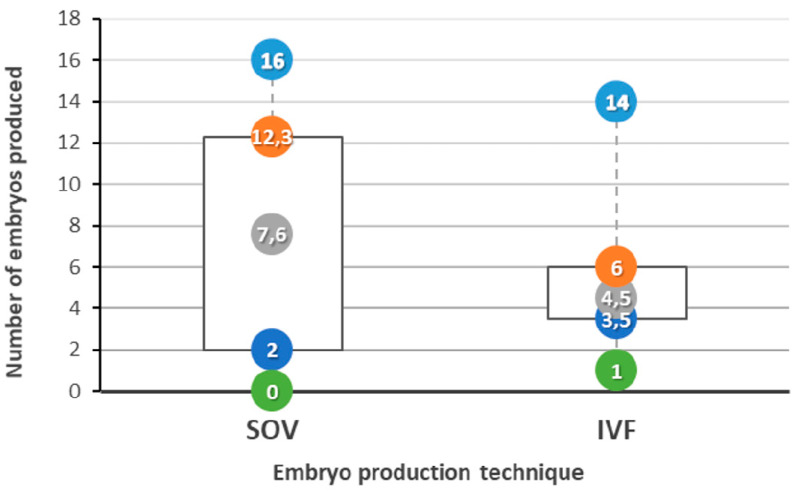
Number of embryos (minimum (green), Q1 (dark blue), average (gray), Q3 (orange) and maximum (light blue)) produced by all the 10 cows in 5 different collections using different techniques SOV and in vitro fertilization (IVF).

The average number of embryos produced by the cows using SOV was 7.63 ± 5.61 (min = 0, max = 16) and 4.52 ± 2.44 by IVF (min = 1, max = 14). A higher variation in the production of SOV embryos was identified when compared to IVF (Figure 2). There was no statistical difference (*p* = 0.303) among the number of embryos produced by IVF or SOV. In addition, no significant correlation was identified between the number of embryos produced by cow and technique (r = 0.379, *p* = 0.529). The total cost of each embryo produced by SOV was R$215.00 and R$410.00 for the IVF (Table 1), which included the veterinary labor, hormones, embryologist work and the semen.

For the IVF costs, the laboratories used had a set price of R$410.00 per embryo produced and transferred.

## 4. Discussion

The main goal of our study was to test the efficacy of a lower total dose of FSH (175 mg) in the SOV protocol to produce in vivo Wagyu embryos. Our protocol proved to very effective for the group of animals used in this study, since they have produced an average of 7.6 embryos per cow per collection, 1.4 embryos more than a study conducted by An et al. 2016 [20], which used greater doses of FSH (400 mg) in a Wagyu population. Although the average number of embryos produced by the SOV technique was higher, we also observed a higher variation among the number of embryos produced by this technique when compared to IVF, even when considering that the IVF technique was conducted by different labs. In 60% of the animals used in our study, we observed a consistently superior response of the SOV protocol (11.38 ± 3.23 embryos) when compared to IVF (5.23 ± 2.28). In the remaining 40% of the animals, we observed a better response to the IVF technique (5 ± 3.2 embryos) when compared to the SOV procedure (2 ± 3.6). This was likely caused by individual genetic variation among animals used in this study, which might have affected their response to the superovulation stimulus. Mutations in the *GRIA1* gene have been described to be involved with superovulation rates in Wagyu cattle [14]. In addition to that, a study conducted by Hirayama and colleagues [15] identified that levels of plasma anti-Müllerian hormone (AMH) concentration, age in months, repeated superovulation, mutations in the *FSHR* gene and bloodlines had significant effects on the responses to superovulation. To minimize the effect of the genetic variation in response to SOV protocols, we used only animals with a greater percentage of the Tajima line.

With the SOV protocol used, we also observed animals that successfully ovulated (based on the presence of corpus luteum) but did not produce embryos, possibly due to the FSH-induced hyper stimulation of ovaries. This demonstrates the need of adjustments in the FSH dose on those animals for future SOV work. This variation was also observed by Mapletoft et al. [23], especially with an increase in the days of the FSH stimulation, which was associated with a superior number of transferable embryos. A study conducted by Du et al. [24] using a low dose of FSH (10 mg) to induce multiple ovulation in Japanese black cattle (Wagyu) obtained an inferior response in the number of embryos produced by SOV (5.8 ± 1.1) and a pregnancy rate of 38.2%. The insemination dose used per cow in the SOV protocol was ten times higher than the sperm number used for the IVF technique. Following a feasibility analysis of protocol type for the production of SOV embryos versus IVF embryos, a clear economic advantage was observed with the SOV protocol; the cost to produce SOV embryos was always lower when compared to the IVF embryos (set price), as long as the superovulated cow produced five or more embryos per collection.

As described by An et al. [20], the hormones administered in a superovulation protocol and embryo transfer protocol are part of the initial upfront costs involved for this technique, which may be either cost-prohibitive or hard to obtain in a developing country. Considering this, the use of the IVF technique is a great alternative to attained high pregnancy rates in animals that do not respond well to SOV protocols; at the same time, that improves the production and the genetic value of Wagyu herds.

## 5. Conclusions

In conclusion, the SOV technique showed a greater outcome for producing Wagyu embryos and presented a lower cost per embryo produced if cows responded well to the protocol, as evidenced by the production of more than five embryos per collection. However, for the cows that produced a lower number of embryos per collection, the SOV protocol was not economically viable for its use. Therefore, it is suggested that the IVF technique could be used instead to reduce the cost per embryo produced of those animals.

## Figures and Tables

**Table 1:** Total cost for embryo production and collection using the superovulation protocol (SOV) technique. R$ values expressed in Reais, and US$ dollar conversion rate is R$5.00 to US$1.00.

Product	Total Dose	Cost (R$)	Cost ($)
CIDR	1.90 mg	20.00	4.00
FSH	165 mg	218.00	43.60
Cloprostenol	1.50 mg	12.00	2.40
Estradiol Benzoate	3.00 mg	1.00	0.20
GnRh	0.05 mg	19.00	3.80
Semen	2	200.00	40.00
AI Service	2	120.00	24.00
Material SOV Collection		60.00	12.00
Labor		980.00	196.00
TOTAL		R$1630.00	$326.00
